# Diagnostic imaging in the management of older adults with low back pain: analysis from the BAck Complaints in Elders: Chiropractic – Australia cohort study

**DOI:** 10.1186/s12998-024-00562-z

**Published:** 2024-12-18

**Authors:** Hazel J. Jenkins, Kristin Grace, Anika Young, Felix Parker, Jan Hartvigsen, Sidney M. Rubinstein, Simon D. French, Katie de Luca

**Affiliations:** 1 Department of Chiropractic, Macquarie University, Sydney, Australia; 2 School of Health, Medical and Applied Sciences, CQUniversity, Rockhampton, Australia; 3 Centre for Muscle and Joint Health, Department of Sports Science and Clinical Biomechanics, University of Southern Denmark, Odense, Denmark; 4 Chiropractic Knowledge Hub, Odense, Denmark; 5 Department of Health Sciences, Faculty of Science, Amsterdam Movement Sciences Research Institute, Vrije Universiteit, Amsterdam, The Netherlands

**Keywords:** Low back pain, Chiropractic, Elderly, Diagnostic imaging

## Abstract

**Background:**

Diagnostic imaging is commonly used in the management of low back pain (LBP), with approximately one-quarter of those who present to primary care referred for imaging. Current estimates of imaging frequency commonly exclude older adults; however, pathology detected with imaging (e.g., osteoporosis, cancer) may occur more frequently in older populations. The aims of this study were to: (i) determine the frequency and forms of diagnostic imaging use in older adults presenting for chiropractic care for LBP in Australia; (ii) describe participant characteristics associated with imaging use; and (iii) describe the types of radiographic findings.

**Methods:**

Data were collected from the BAck Complaints in Elders: Chiropractic-Australia (BACE: C-A) study, a 12-month, prospective cohort study of adults aged ≥ 55 years with a new episode of LBP. Self-reported frequency of imaging use (baseline, 2 and 6 weeks, 3, 6, 9, and 12 months) was reported descriptively by imaging modality. Imaging reports were obtained, and imaging findings were independently extracted and categorised. Baseline characteristics were assessed for differences in those who received imaging compared to those who did not. Proportions of imaging use and imaging findings were presented descriptively with 95% confidence intervals.

**Results:**

The BACE: C-A cohort comprised 217 participants of whom 60.8% reported receiving diagnostic imaging for their current episode of LBP. X-ray was performed most (44.7%), followed by computed tomography (CT) (30.8%). Participants receiving imaging reported higher low back disability, more healthcare use for LBP, more frequent leg pain, more suspected serious pathology, and stronger beliefs that imaging was important. Degenerative changes were the most common imaging finding (96.6%). Pathology of possible clinical significance, including compression fracture or suspected osteoporosis, was present in 15.5% of participants.

**Conclusion:**

Three out of five older adults with LBP who sought chiropractic care received imaging over one-year. Participants receiving imaging tended to have more complex presentations (e.g., more disability, suspected underlying pathology) or stronger beliefs that imaging was necessary for the management of LBP. Degenerative changes were the most common imaging finding. Pathology of potential clinical relevance was present on approximately 15% of imaging reports received. No conditions requiring immediate medical attention were reported.

**Supplementary Information:**

The online version contains supplementary material available at 10.1186/s12998-024-00562-z.

## Background

Low back pain (LBP) is the leading cause of years lived with disability worldwide, with peak disability burden found in the 80- to 84-year age group [1]. In 2017, the age-specific point prevalence of LBP in older adults ranged between 14.7% in males aged 65- to 69-years to 22.7% in females aged 80- to 89-years [2, 3]. Among older adults, 60% have LBP-related functional disability, which substantially limits their ability to engage in activities of daily living and reduces mobility [4, 5].

Diagnostic imaging is commonly used in the management of LBP, with approximately one-quarter of people who present to primary care with LBP referred for imaging [6]. Imaging is recommended to rule out underlying serious pathology (e.g., tumour, infection, fracture), or recommended when surgical management is considered [7–9]. Current estimates of the frequency of imaging commonly exclude older adult populations. In a previous review of imaging use for LBP [6], only two of the 45 included studies assessed the proportion of older adults referred for imaging [10, 11], with proportions of imaging in the first four weeks varying between 28.8% [10] and 38.3% [11].

Clinical practice guidelines recommend that imaging for LBP is primarily indicated if there are signs or symptoms of possible underlying pathology, or ‘red flags’, or where there is a lack of improvement in the presenting condition after four to six weeks of appropriate management [8, 9, 12]. Early imaging in older adults without suspected underlying pathology has been associated with poorer outcomes and increased downstream healthcare use [13, 14]. In older guidelines, age over 50 by itself was considered a red flag for possible serious pathology [15, 16]; however, more recent recommendations consider older age within a group of risk factors for fracture and not a red flag by itself [7, 17]. Variability in imaging use in older adults is high, potentially indicating clinician uncertainty of when imaging is needed. For example, in one study, individual clinicians referred between 6% and 54% of older adults with LBP for imaging [11].

In chiropractic practice, one in seven adult patients are over 65 years of age and of these patients, nearly 56% present with a back problem [4]. Controversy related to the appropriate use of diagnostic imaging for LBP practice has been reported in chiropractic practice [8]. In contrast to LBP clinical guideline recommendations, some chiropractors report using imaging routinely to assess for contraindications to spinal manipulation or perform biomechanical analysis [18]. High velocity low amplitude (HVLA) manipulation is the most common form of treatment to be performed by chiropractors, but it is selected as a treatment less often in older patients [19]. HVLA manipulation is used in only 60% of chiropractic encounters with patients aged 65 years or over, compared to 92% of encounters with younger patients [4], possibly reflecting a perceived increased risk of harm due to an underlying pathology, with conditions such as osteoporosis (and related fractures) and tumours more likely to occur in older adult populations [20, 21]. Arguments have been made, therefore, that intention to use HVLA manipulation may increase the need for diagnostic imaging to assess for underlying pathology that would contraindicate manipulation [8]. Previous studies in cohorts of chiropractic patients have found relatively low incidence of serious pathology or anomalies that would be likely to contraindicate manual therapy [8, 22–25]; however, these studies did not specifically assess older adults.

The overall aim of this study was to assess the use of imaging in older adults with a new episode of LBP who sought chiropractic care and to describe the types of radiographic findings. The specific aims were to:

(i) determine the proportion of older adults with LBP presenting for chiropractic care who reported receiving low back imaging across the 12-month follow-up period and describe the type and timing of imaging received;
(ii) describe participant characteristics associated with receiving imaging; and.
(iii) describe the types of imaging findings in older adults receiving imaging of the low back.

## Methods

### Study design

Data were collected in the BAck Complaints in Elders – Chiropractic: Australia (BACE: C-A) study [26], a 12-month longitudinal cohort study of older adults with LBP presenting for chiropractic care in Australia. Ethics approval was provided by Macquarie University, Approval No.: 5201954609164. This study was reported in accordance with the STROBE reporting guidelines for cohort studies [27].

### BACE: C-A cohort study

The BACE: C-A study was conducted from October 2019 to November 2022, as reported previously [26]. Twenty-eight chiropractors were recruited through national chiropractic conferences and notices in professional magazines. Chiropractors had a spread of characteristics including practice location, years in practice, and average number of patients seen per week (Additional file Table 1). Compared to chiropractors across Australia [28], recruited chiropractors had similar demographic characteristics except for practice location, with 43% practicing in New South Wales and none in South Australia, Northern Territory, or Australian Capital Territory. Most of the chiropractors (89%) referred for radiographs at medical radiology centres and would occasionally refer people of 55 years or over for radiographic imaging (61%). Less than one-third (29%) of chiropractors thought that routine radiographs were necessary before initiating spinal manipulative therapy in people of 55 years or over, similar to results from a previous survey of Australian chiropractors [18].

Patients were informed about the study procedures on-site at the chiropractic clinic, prior to the consultation with the recruited chiropractor for their back pain. Interested patients were screened by the chiropractic receptionist or the study research assistant, using pre-determined eligibility criteria [26], and if eligible gave informed consent in person or online, dependent on the participant’s preference. Participants were included if they were aged 55 years or older and presented to a chiropractor for a new episode of LBP, defined as either the first episode of LBP or an episode where the person had not sought care with a chiropractor in the preceding 6-months. LBP was defined as pain between the 12th ribs and the bottom of the buttock, with or without leg pain. Participants were excluded at baseline if a serious cause of LBP (e.g., tumour, fracture, infection) was diagnosed, a contraindication for chiropractic care was suspected, or if they were unable to complete online questionnaires due to language or computer literacy restrictions. Only people contraindicated for all forms of chiropractic management were excluded. People contraindicated for a specific treatment option (e.g., HVLA manipulation), but still able to receive other forms of chiropractic management were eligible for inclusion. All participants meeting inclusion criteria and consenting to participate in the BACE: C-A study were included in the current analysis.

Participants were asked to complete a baseline questionnaire either before or directly after their first chiropractic visit. Follow-up questionnaires were completed at two- and six-weeks and three-, six-, nine-, and 12-months. The questionnaires collected information on sociodemographic characteristics, LBP characteristics, medical history, healthcare use, imaging use, and a range of psychosocial and lifestyle characteristics.

Participants who self-reported receiving imaging at any of the timepoints were asked for consent to provide the associated imaging reports to the research team. Imaging reports were anonymised by an independent research assistant. The blinded reports were extracted independently by two other research assistants, supervised by one of the research team (HJ). Where discrepancies were identified, the extracted data were compared and corrected to the original report. Extracted data included date of imaging, type and region of imaging, and the imaging findings (as written in the report). The original images were not requested or assessed.

### Outcome measures

The use of imaging was assessed by self-report in each of the baseline and follow-up questionnaires. In the baseline questionnaire, participants were asked ‘Before your visit with the chiropractor today, have you had an x-ray or any other imaging or test (e.g. computed tomography (CT) scan, magnetic resonance imaging (MRI), bone scan) for THIS episode of back pain?’ Imaging reported at baseline could include imaging received prior to or during the first visit with the chiropractor. In subsequent questionnaires, participants were asked if they had received imaging since the previous questionnaire. If yes, they were asked to describe the imaging type/s received and the type of practitioner who had referred them for the imaging (chiropractor, general medical practitioner, physiotherapist, other).

Participant characteristics considered likely to be associated with use of imaging were selected through discussion within the authorship team, informed by results of previous studies [29, 30] and imaging guideline recommendations [31]. Extracted participant characteristics included self-reported data on sociodemographic characteristics: age (years), gender (male/female/unspecified); clinical characteristics: LBP intensity in the last week (0–10 scale), presence of pain extending into the lower leg below the knee or into the foot (yes/no), duration of LBP (less than 6-weeks, 6-weeks to 3-months, more than 3-months), previous episode of LBP (yes/no), low back disability (24 point Roland-Morris Disability Questionnaire), previous other health care for the current LBP presentation (yes/no), potential serious cause of LBP (practitioner reported that back pain is caused by any of fracture, arthritis, cancer, infection; yes/no), past history of cancer (yes/no), taking glucocorticoid medication (yes/no); and beliefs about the importance of imaging (5-point Likert scale: agree (4 or 5)/neutral [3]/disagree (1 or 2) with 2 questions: ‘X-rays or scans are necessary to get the best medical care for LBP’; and ‘Everyone with LBP should have spine imaging, e.g. X-ray, CT, or MRI’).

Imaging findings were synthesised into the following diagnostic categories by two independent researchers (HJ and KG): congenital, spondylolisthesis, alignment changes (scoliosis and other), arthritis (degenerative and inflammatory), disc lesions (disc bulge, protrusion, herniation, sequestration, annular tear), ligamentum flavum hypertrophy, spinal stenosis (central or lateral recess), modic changes or bone oedema (types I, 2, 3, and bone oedema), trauma (fracture and new or old vertebral body compression), osteoporosis (osteopenia, possible osteoporosis), serious pathology (tumours, infection), soft tissue changes, and evidence of prior surgery. Reported imaging findings categorised into trauma (new fractures only), osteoporosis, and serious pathology were combined into an additional category: ‘pathology or trauma of likely clinical significance’, as these conditions would commonly contraindicate HVLA manipulation or require referral for medical assessment. Any discrepancy in categorisation was resolved through discussion.

### Data analysis

The proportion of participants who received imaging was reported as: (i) the proportion reporting receiving at least one type of imaging across the 12-month period; (ii) the proportion receiving at least one type of imaging at each of the questionnaire timepoints; (iii) the proportion receiving each imaging type (x-ray, CT, MRI, other) across the 12-month period and at each time point; and (iv) the proportion reporting their first imaging across the 12-month study period at each time point. Proportions were presented descriptively as a percentage with 95% confidence intervals (95% CIs). No data imputation was performed; participants who did not respond to the imaging question were assessed as not receiving imaging at that timepoint.

Participant characteristics were reported descriptively using means and standard deviations for continuous variables (age, pain intensity, disability) and proportions with 95% CIs for categorical variables (gender, duration of back pain, previous episode of back pain, previous health care, possible serious cause of LBP, history of cancer, taking glucocorticoid medication, imaging beliefs). Characteristics were reported for the total sample and separately for those who did and who did not receive at least one type of imaging across the 12-month period. Statistically significant differences in characteristics between the groups who received imaging and those who did not were assessed using the Welch two-sample t-test for continuous variables and the Fisher’s exact test for categorical variables.

Types of imaging findings among participants were reported as proportions (percentage with 95% CIs) with three different denominators: (i) the number of participants who provided at least one imaging report; (ii) the number of imaging reports in total (some participants provided more than one imaging report); and (iii) the number of imaging reports stratified by the type of imaging (x-ray, CT, MRI, bone scan, dual-energy x-ray absorptiometry (DXA)).

## Results

**Fig. 1:**
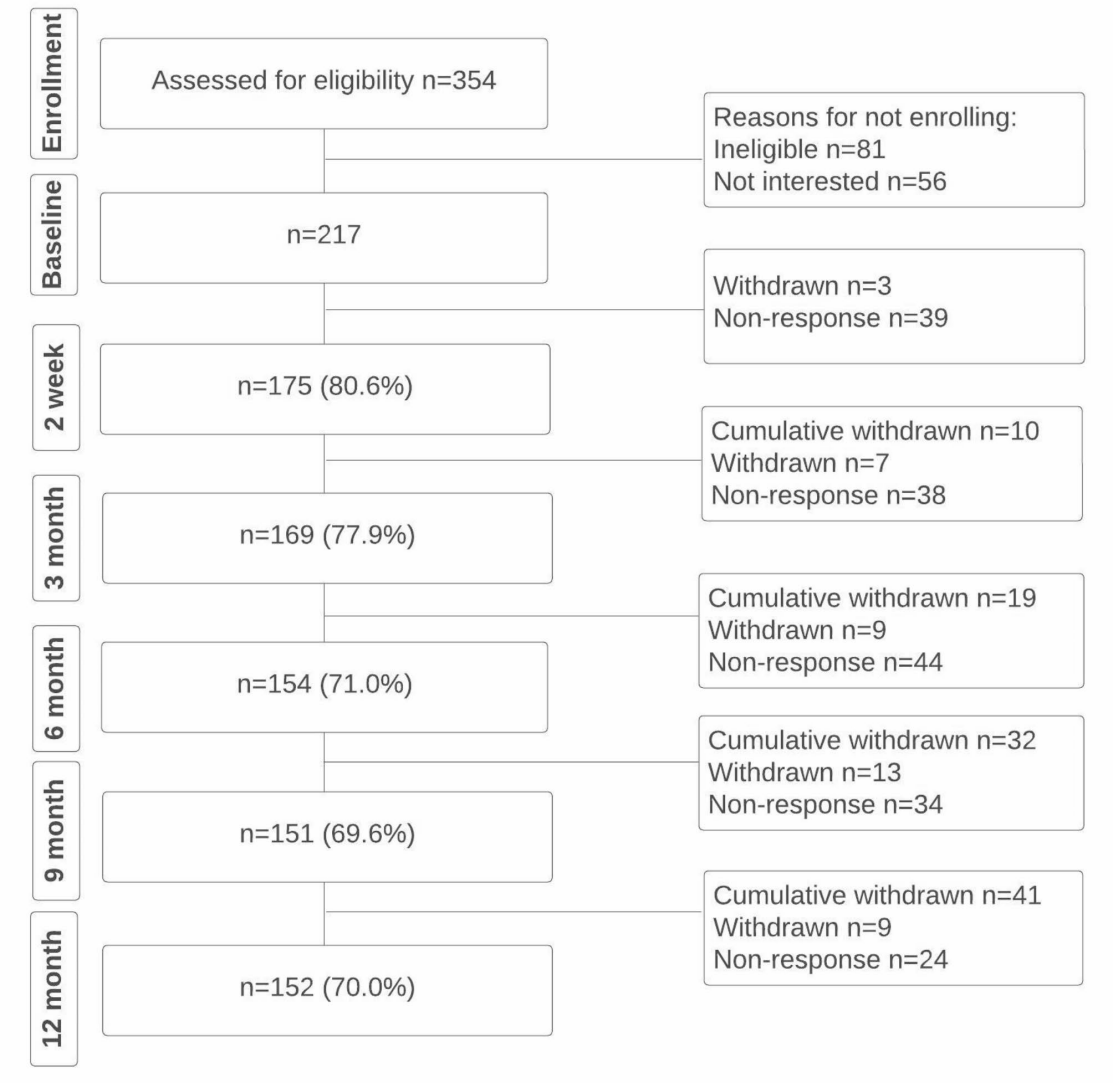
Flow of participants through the study. Reasons for withdrawal: Too time-consuming *n* = 8; Low back pain resolved and didn’t wish to continue *n* = 2; Not interested in participating any more *n* = 5; Family issues *n* = 2; Medical issues *n* = 10; Translation issue *n* = 1; No reason provided *n* = 13

The BACE: C-A cohort recruited 217 older adults, all of whom were included in this analysis (Fig. 1). Over the course of the study 41 participants withdrew; however, data that had already been collected for these participants remained in the study. Two people were identified as having a serious cause of LBP and excluded from the study. There was a mean age of 67.4 years (SD: 7.8) and 50.9% (95%CI: 44.3–57.6) were female (Table 1).

**Table 1:** Baseline characteristics of the participants in the BACE: C-A cohort (*n* = 217), stratified by those that reported receiving (*n* = 132) and not receiving (*n* = 85) imaging

Characteristic	All participants (*n* = 217)	Participants receiving imaging (*n* = 132)	Participants not receiving imaging (*n* = 85)
Age (mean, SD)	67.4, 7.8	67.2, 7.6	67.7, 8.1
Sex (n, %, 95%CI)			
Male	104, 49.1, 42.4–55.7	68, 52.3, 43.8–60.7	36, 43.9, 33.7–54.7
Female	108, 50.9, 44.3–57.6	62, 47.7, 39.3–56.2	46, 56.1, 45.3–66.3
Low back pain intensity (mean, SD)	5.7, 2.4	5.7, 2.3	5.6, 2.6
Duration of low back pain (n, %, 95%CI)			
Less than 6 weeks	106, 49.5, 42.7–56.4	61, 46.6, 37.9–55.5	45, 54.2, 43.0-65.1
6 weeks to 3 months	27, 12.6, 8.6–18.0	17, 13.0, 8.0-20.2	10, 12.0, 6.2–21.5
More than 3 months	81, 37.9, 31.4–44.7	53, 40.5, 32.1–49.4	28, 33.7, 24.0–45.0
Low back disability (mean, SD)*	6.6, 5.3	7.2, 5.1	5.7, 5.5
Previous low back pain (n, %, 95%CI)	183, 85.1, 79.7–89.3	115, 87.8, 81.1–92.3	68, 81.0, 71.3–87.9
Previous healthcare for low back pain (n, %, 95%CI)*	105, 48.4, 41.6–55.2	75, 56.8, 47.9–65.3	30, 35.3 25.4–46.5
Pain extending below the knee (n, %, 95%CI)*	40, 18.4, 13.6–24.4	30, 22.7, 16.1–31.0	10, 11.8, 6.1–21.0
Possible serious cause of low back pain (n, %, 95%CI)*	68, 31.3, 25.3–38.0	57, 43.2, 34.7–52.1	11, 12.9 6.9–22.4
Past history of cancer (n, %, 95%CI)	23, 13.9, 9.4–19.9	15, 15.5, 9.6–24.0	8, 11.6, 6.0-21.2
Taking glucocorticoid medication (n, %, 95%CI)^#^	5, 2.4, 1.0-5.6	3, 2.3, 0.8–6.6	2, 2.6, 0.7–9.1
X-rays or scans are necessary to get the best medical care for low back pain (n, %, 95%CI)*			
Agree	126, 61.0, 53.8–67.5	90, 69.2, 60.4–76.9	36, 46.8, 35.4–58.4
Neutral	52, 25.1, 19.5–31.7	27, 20.8, 14.4–29.0	25, 32.5, 22.5–44.2
Disagree	29, 14.0, 9.7–19.7	13, 10.0, 5.6–16.8	16, 20.8, 12.7–31.8
Everyone with low back pain should have spine imaging (e.g. X-ray, CT, or MRI)			
Agree	109, 52.7, 45.9–59.8	74, 56.9, 48.0-65.5	35, 45.5, 34.2–57.2
Neutral	61, 29.5, 23.5–36.3	37, 28.5, 21.1–37.2	24, 31.2, 21.4–42.9
Disagree	37, 17.9, 13.0-23.9	19, 14.6, 9.3–22.1	18, 23.4, 14.8–34.7

*statistically significant difference in this variable between those that received imaging and those that did not, *p* < 0.05; ^#^p-value not reported, data too sparse

Across the 12-month follow-up period, 132/217 (60.8%, 95%CI: 54.0-67.4) participants received at least one type of imaging for their new episode of LBP (Table 2). These 132 participants reported receiving 226 diagnostic imaging studies (mean: 1.7 per participant), with 68.2% (95%CI: 59.4–75.9) of participants referred for imaging by chiropractors and 47.0% (95%CI: 83.3–55.8) by general medical practitioners (Additional file Table 2). Approximately half of the participants reported receiving initial imaging either during, or prior to, their first visit with the chiropractor (23.7%, 95%CI: 18.4–30.2) or between 3 and 6 months after their first visit (22.6%, 95%CI: 17.3–28.8). X-ray was the most frequent imaging type (44.7%, 95%CI: 37.0-51.6), followed by CT scans (30.8%, 95%CI: 24.9–37.6%), and MRI (13.82%, 95%CI: 9.7–19.3). Other imaging was performed in 14.8% of participants (95%CI: 10.5–20.3) and included bone scans, DXA, and diagnostic ultrasound.

**Table 2:** Use of imaging amongst the 217 participants across the 12-month study period, stratified by imaging type and time-period (n, %, 95%CI)

	Any imaging type	First report of any imaging	X-ray	CT	MRI	Other*
Any imaging	132, 60.8, 54.0-67.4	Not applicable	97, 44.7, 37.0-51.6	67, 30.8, 24.9–37.6	30, 13.82, 9.7–19.3	32, 14.8, 10.5–20.3
Baseline	52, 23.7, 18.4–30.2	52, 23.7, 18.4–30.2	40, 18.4, 13.8–24.1	24, 11.1, 7.5–15.9	14, 6.5, 3.9–10.5	12, 5.5, 3.2–9.4
2-weeks	13, 6.0, 3.2–10.0	9, 4.1, 2.0–8.0	9, 4.1, 2.2–7.7	1, 0.5, 0.1–2.6	1, 0.5, 0.1–2.6	2, 0.9, 0.3–3.3
6-weeks	19, 8.8, 5.3–13.3	8, 3.7, 1.7–7.4	11, 5.1, 2.9–8.8	2, 0.9, 0.3–3.3	4, 1.8, 0.7–4.6	4, 1.8, 0.7–4.6
3-months	10, 4.5, 2.2–8.3	6, 2.8, 1.1–6.2	4, 1.8, 0.7–4.6	3, 1.4, 0.5-4.0	4, 1.8, 0.7–4.6	1, 0.5, 0.1–2.6
6-months	68, 31.1, 25.2–37.9	49, 22.6, 17.3–28.8	40, 18.4, 13.8–24.1	37, 17.1, 12.6–22.6	7, 3.2, 1.6–6.5	11, 5.1, 2.9–8.8
9-months	16, 7.4, 4.3–11.7	4, 1.8, 0.6-5.0	9, 4.1, 2.2–7.7	7, 3.2, 1.6–6.5	4, 1.8, 0.7–4.6	3, 1.4, 0.5-4.0
12-months	15, 6.9, 3.9–11.1	4, 1.8,0.6-5.0	6, 2.8, 1.3–5.9	7, 3.2, 1.6–6.5	2, 0.9, 0.3–3.3	6, 2.8, 1.3–5.9

*Other includes bone scan, DXA, diagnostic ultrasound

At baseline, participants who received imaging compared to those who did not receive imaging tended to have higher low back disability (mean 7.2, SD: 5.1 versus 5.7, SD: 5.5), higher previous healthcare use for LBP (56.8%, 95%CI: 47.9–65.3 versus 35.3%, 95%CI: 25.4–46.5), more frequent pain extending below the knee (22.7%, 95%CI: 16.1–31.0 versus 11.8%, 95%CI: 6.1–21.0), had been told more frequently that there was a potential serious cause to their LBP (43.2%, 95%CI: 34.7–52.1 versus 12.9%, 95%CI: 6.9–22.4), and had more frequent beliefs that imaging is necessary for the management of LBP (69.2%, 95%CI: 60.4–76.9 versus 46.8%, 95%CI: 35.4–58.4) (Table 1).

**Fig. 2:**
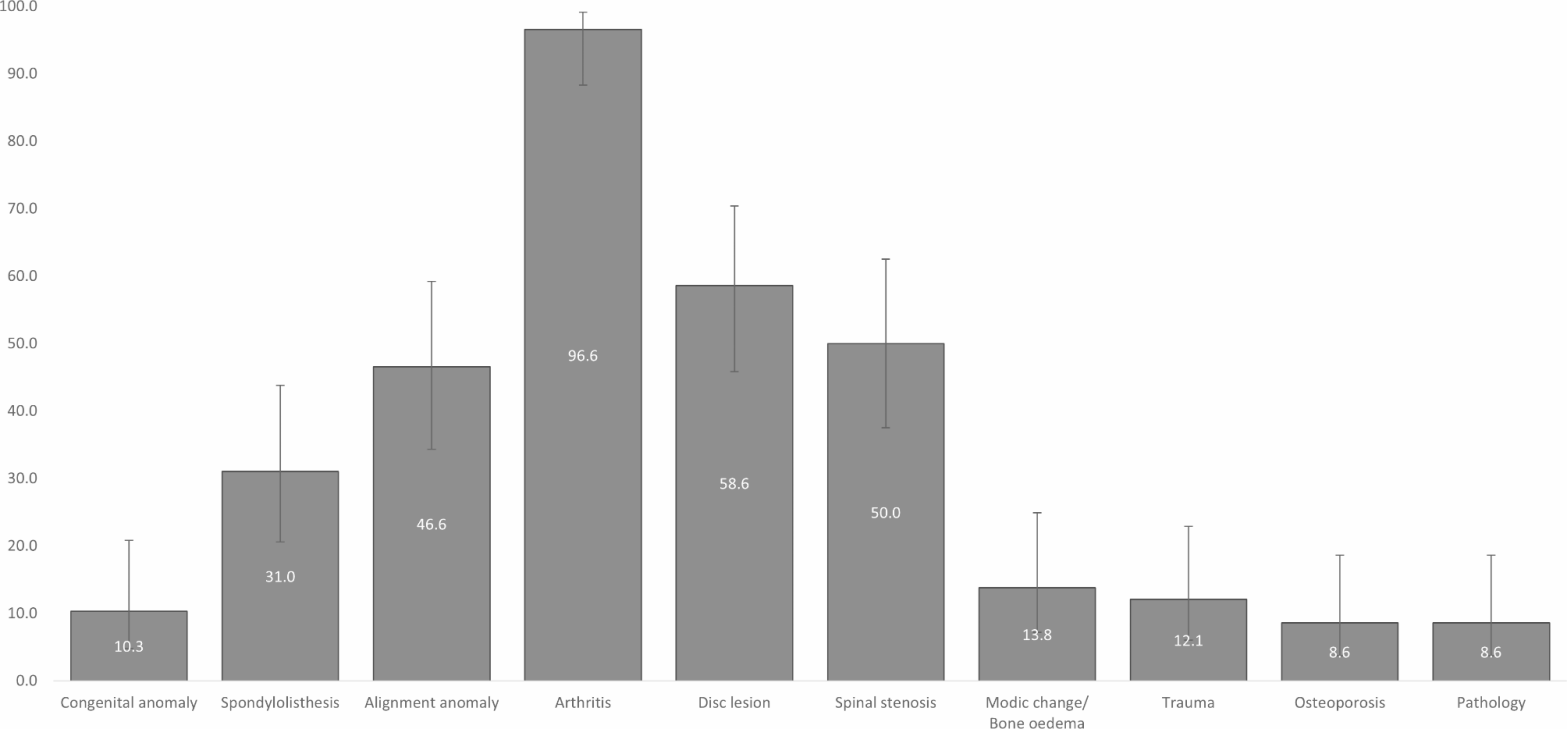
Proportion of imaging findings (%), categorised by pathology/lesion type, for all participants providing imaging reports (*n* = 58)

Of those who received imaging, 58/132 (43.9%, 95%CI: 35.8–52.5) provided imaging reports of the lumbar spine, including 29 X-ray, 22 CT, 15 MRI, two bone scan, and three DXA reports (total 71 reports, mean 1.2 reports per participant). The sociodemographic and clinical characteristics of participants who provided their imaging reports were similar to those who did not, except for LBP intensity and duration (Additional file Table 3). Arthritis was the most common condition in the imaging reports (96.6%, 95%CI: 88.3–99.1), with almost all reports related to degenerative changes (94.8%, 95%CI: 85.9–98.2) and one report of diffuse idiopathic skeletal hyperostosis (DISH). There were no reports of inflammatory arthritis. All CT and MRI reports identified both degenerative arthritis (disc and /or facet degeneration) and varieties of disc lesions (disc bulge, herniation, or annular tear/fissure). Spinal stenosis (central or lateral recess) was reported in 77.3% (95%CI: 56.6–89.9) of CT reports and 93.3% (95%CI: 70.2–98.8) of MRI reports. Alignment changes, mainly scoliosis, were reported in 46.6% (95%CI: 34.3–59.2) of participants who provided imaging reports. Pathology or trauma of likely clinical significance was present in 9/58 (15.5%, 95%CI: 8.4–26.9) including one new osteoporotic vertebral compression fracture, five reports of possible osteoporosis or osteopenia, three haemangiomas, two Tarlov cysts, and one femoral neck bone infarct. No cases of new traumatic fracture, spinal infection or cancer were found (Table 3; Fig. 2, and Additional file Table 4).

**Table 3:** Proportion of imaging findings categorised by pathology/lesion type and stratified by the individual and the type of imaging report (n, %, 95%CI)

	Findings per individual	Findings per imaging report	Findings per x-ray	Findings per CT	Findings per MRI	Findings per bone scan	Findings per DXA
Congenital anomaly	6, 10.3, 4.8–20.8	7, 9.9, 4.9–19.0	1, 3.5, 0.6–17.2	4, 18.2, 7.3–38.5	2, 13.3, 3.7–37.9	0, 0.0, 0.0-65.8	0, 0.0, 0.0-56.2
Spondylolisthesis	18, 31.0, 20.6–43.8	20, 28.2, 19.0-39.5	3, 10.3, 3.6–26.4	7, 31.8, 16.4–52.7	10, 66.7, 41.7–84.8	0, 0.0, 0.0-65.8	0, 0.0, 0.0-56.2
Alignment anomaly	27, 46.6, 34.3–59.2	30, 42.3, 31.5–53.9	14, 48.3, 31.4–65.6	10, 45.5, 26.9–65.3	6, 40.0, 19.8–64.3	0, 0.0, 0.0-65.8	0, 0.0, 0.0-56.2
Arthritis*	56, 96.6, 88.3–99.1	65, 91.6, 82.8–96.1	26, 89.7, 73.6–96.4	22, 100.0, 85.1–100.0	15, 100.0, 79.6–100.0	2, 100.0, 34.2, 100.0	0, 0.0, 0.0-56.2
Disc lesion	34, 58.6, 45.8–70.4	37, 52.1, 40.7–63.3	0, 0.0, 0.0-11.7	22, 100.0, 85.1–100.0	15, 100.0, 79.6–100.0	0, 0.0, 0.0-65.8	0, 0.0, 0.0-56.2
Ligamentum flavum hypertrophy	10, 17.2, 9.6–28.9	10, 14.1, 7.8–24.0	0, 0.0, 0.0-11.7	5, 22.7, 10.1–43.4	5, 33.3, 15.2–58.3	0, 0.0, 0.0-65.8	0, 0.0, 0.0-56.2
Spinal stenosis^$^	29, 50.0, 37.5–62.5	32, 45.1, 34.1–56.6	1, 3.5, 0.6–17.2	17, 77.3, 56.6–89.9	14, 93.3, 70.2–98.8	0, 0.0, 0.0-65.8	0, 0.0, 0.0-56.2
Modic change/ Bone oedema	8, 13.8, 7.2–24.9	8, 11.3, 5.8–20.7	0, 0.0, 0.0-11.7	1, 4.5, 0.8–21.8	7, 46.7, 24.8–69.9	0, 0.0, 0.0-65.8	0, 0.0, 0.0-56.2
Trauma	7, 12.1, 6.0-22.9	8, 11.3, 5.8–20.7	2, 6.9, 1.9–22.0	3, 13.6, 4.8–33.3	3, 20.0, 7.1–45.2	0, 0.0, 0.0-65.8	0, 0.0, 0.0-56.2
Osteoporosis	5, 8.6, 3.7–18.6	6, 8.5, 3.9–17.2	1, 3.5, 0.6–17.2	3, 13.6, 4.8–33.3	0, 0.0, 0.0-20.4	0, 0.0, 0.0-65.8	2, 66.7, 20.8–93.9
Serious pathology^#^	5, 8.6, 3.7–18.6	5, 7.0, 3.1–15.5	1, 3.5, 0.6–17.2	2, 9.1, 2.5–27.8	2, 13.3, 3.7–37.9	0, 0.0, 0.0-65.8	0, 0.0, 0.0-56.2
Pathology/trauma of likely clinical significance	9, 15.5, 8.4–26.9	10, 14.1, 7.8–24.0	2, 6.9, 1.9–22.0	4, 18.2, 7.3–38.5	2, 13.3, 3.7–37.9	0, 0.0, 0.0-65.8	2, 66.7, 20.8–93.9

*Arthritis included degenerative conditions and inflammatory arthritis. No inflammatory arthritis was identified. See Additional file Table 4 for more detail

^$^Spinal stenosis includes central canal stenosis and lateral recess stenosis. See Additional file Table 4 for more detail

^#^Pathology included three haemangiomas, two Tarlov cysts, and one femoral neck bone infarct. One participant had both a haemangioma and a Tarlov cyst

## Discussion

In a cohort of 217 people aged ≥ 55 years who sought chiropractic care for a new episode of LBP, 132 (60.8%) reported receiving diagnostic imaging either prior to their first consult with the chiropractor or within the 12-month follow-up period. Approximately two-thirds (68.2%) of participants were referred for imaging by a chiropractor. X-rays (44.7%) were the most common imaging modality received, followed by CT (30.8%) and MRI (13.8%), with the majority receiving between one and two different forms of imaging. Participants who received imaging tended to have higher low back disability, more previous healthcare use for LBP, more frequent pain extending below the knee, more suspected serious pathology, and beliefs that imaging is necessary for the management of LBP.

Degenerative change was the most common imaging finding reported, present in 94.8% of participants who provided imaging reports. All participants who received a CT scan or MRI had degenerative change (disc and/or facet) and one or more disc lesions (disc bulge, herniation, or annular tear/fissure). 15% of people providing imaging reports had underlying pathology or trauma of likely clinical significance, including osteopenia/possible osteoporosis or benign bone tumours. No conditions requiring immediate or urgent medical referral, such as infection, cancer, or new traumatic fractures were identified.

### Comparison to previous literature

Previous studies assessing imaging of the low back in older adults presenting for medical care found that between 28.8% [10] and 38.3% [11] of participants received imaging within the first 4 weeks. This is comparable to the present study where 23.7% of participants received imaging prior to or at their first consult with the chiropractor, and by six weeks 31.8% had received imaging. Results are also comparable to a meta-analysis of 8 studies in primary care (medical, chiropractic, physiotherapy, osteopathy), which found the proportion of low back imaging in adults of any age presenting for care to be 24.8% (95%CI: 19.3-31.1%) [6]. In the current study, we found that an additional 22.6% of participants received their first diagnostic imaging between three and six months; however, this was not seen in a previous study of older adults presenting for medical care where total imaging only increased from 28.8 to 32.2% (increase of 3.4%) between one to six months [10]. Increased imaging after three months in our study may reflect guideline recommendations for ‘watchful waiting’ [8] or a trial of care [9], or the reassessment of persistent or recurrent LBP. However, the reasons for imaging use, and whether they aligned with guideline recommendations for each participant, were not assessed in this study.

The characteristics of those who received imaging were broadly consistent with clinical practice guideline recommendations, including suspected underlying pathology and leg pain extending below the knee [7–9]. Participant beliefs about the importance or necessity of imaging have previously been shown to be a potential driver of increased imaging use [30], consistent with the results of this study. However, the proportion of participants agreeing that imaging is necessary to assess LBP was higher in this study (61.0%) compared to previous studies, performed in chiropractic and medical settings, ranging between 41.5% and 54.3% [29, 30]. Older age in a general adult population of people presenting for medical care has been associated with increased beliefs in the importance of imaging for LBP and may explain the higher proportion of people believing that imaging was needed seen in this study [29].

The types of imaging findings observed in this study are generally consistent with previous literature. Degenerative changes in the lumbar spine, seen in nearly all participants providing imaging reports, are well recognised to be frequently observed on diagnostic imaging, especially as people age [32], and to be more common in people with LBP [33, 34]. Previous studies have only assessed the types of imaging findings in adults of any age, including findings on lumbar radiographs in chiropractic settings [22, 24, 25] and findings on MRI in medical settings [20, 21, 35, 36]. The current study found higher proportions of spondylolisthesis and spinal stenosis, possibly due to an increase in these conditions with older age [20]. In contrast, while low proportions have been reported in previous studies [20, 24, 35], no recent fractures, cancer, or spinal infections were identified. The potential to detect serious pathology was limited in this study by the exclusion of people with a diagnosis of serious pathology or contraindications to chiropractic care at baseline from the BACE: C-A cohort and the low number of imaging reports assessed. The exclusion criteria reflect clinical practice in that people diagnosed with serious pathology or contraindications that are outside the scope of chiropractic care at initial assessment would likely be referred directly for medical management [37]. People presenting for medical care also tend to have poorer general health than people presenting for chiropractic care [38]; it is likely that people in poorer health, and more likely to have serious pathology, would self-select to present to medical rather than chiropractic care for assessment.

### Strengths and limitations

The strengths of this study include the recruitment of a representative inception cohort to assess the use of low back imaging for older adults who sought chiropractic care for their LBP [4, 39]. We specifically included outcome measures to capture imaging data across the 12-month study period and provide detailed data on the use of imaging in this cohort.

Limitations include study exclusion criteria, self-reported data, participant withdrawal, and the number and type of imaging reports received from participants, which impact the generalisability and validity of results. People with a diagnosed pathology (e.g., cancer, infection) or contraindications for chiropractic care were excluded from the study, potentially resulting in an underestimation of imaging use. However, if pathology was previously diagnosed, or contraindications for chiropractic care were identified in clinical practice, it is unlikely that the chiropractor would refer the patient for imaging, as a medical referral for appropriate management would be indicated instead [37]. Only two people were excluded due to diagnosed pathology; therefore, the impact on results is minimal. Recall bias is a limitation of self-reported data and participants may have reported the same imaging event across multiple questionnaires. To mitigate this, imaging use was calculated as any imaging per participant, first imaging per participant, and type of imaging per participant, and trends in patterns of imaging use in the 12-month study period are consistent across the different analysis methods. Over the study period, 41/217 (18.9%) withdrew from the study, with short-term follow-up at two weeks of 80.6% (175/217) and long-term follow-up at 12-months of 70.0% (152/217). Less than 50% of participants who reported receiving imaging provided imaging reports (59/132). The lower response rate for this aspect of the study may have been due to the need to provide additional consent, inability to contact the participant, or lack of availability of the imaging report. While statistically significant differences between those that provided imaging reports and those that did not were only seen for LBP intensity and pain duration, potentially relevant differences between groups were noted for previous healthcare use, possible serious cause of LBP, past history of cancer, and beliefs in the need for low back imaging. The relatively low sample size of imaging reports assessed, exclusion of people with diagnosed pathology or contraindications to chiropractic care, and the low prevalence of serious pathology in the low back (e.g., cancer, infection) [20, 35, 36] means it is unlikely that serious pathology would be detected in this study.

Categorisation of imaging findings may have been inconsistent due to variations in imaging reporting, potentially impacting the validity of results. The images were not requested or assessed by the research team and the imaging reports provided were completed by a variety of radiologists or chiropractors depending on where the imaging was obtained. The imaging reports, therefore, were not reported to a common structure and variation in reporting style may have impacted categorisation. For example, degenerative changes were reported for nearly all participants; however, degenerative scoliosis was only reported in 16.7% of participants with scoliosis. This may not reflect the true incidence of degenerative scoliosis, but rather that some reports may have reported on scoliosis and degeneration separately, possibly due to an inability to determine the relationship between the degenerative changes and the scoliosis without further clinical information.

### Implications for clinical practice and research

Nearly all imaging reports assessed had at least one type of degenerative change present; however, clinical relevance is uncertain. Degenerative changes are expected in an older populations, are commonly found in asymptomatic populations [32], but are also more common in people with LBP [33, 34] and have been associated with worse long-term pain and disability [40]. The presence of degenerative change on imaging does not generally indicate a change in treatment approach, and no specific interventions have been identified as more effective when degenerative changes are present [41]. Therefore, identifying degenerative changes on imaging has limited impact on patient management. Similarly, disc lesions (including disc bulges and disc herniation) were present on all people who received a CT or MRI. While only people with clinical suspicion of a disc lesion may have been referred for CT or MRI, imaging may be of less clinical relevance unless a change in treatment, such as surgery, is being considered [7, 8, 41]. Types of imaging findings that are likely to result in a change to chiropractic management include recent trauma, bone weakening conditions (e.g., osteoporosis, benign tumours) or serious pathology (e.g., cancer, infection) [37]. In this study 15.5% of people providing imaging reports had a bone weakening condition (osteoporosis, haemangioma) that would contraindicate joint manipulation or a condition potentially requiring medical referral (Tarlov cyst, bone infarct) that may be important to identify clinically.

A larger cohort of older adults with data-linkage or routine administrative collection of imaging use and reports (instead of self-report) would allow inclusion of consecutive patients, thus improving the generalisability of results. A larger cohort with routine data collection would also improve the ability to stratify results into age groups and sex and identify types of imaging findings on all participants receiving imaging, including those with underlying serious pathology. In this study we did not attempt to identify the appropriateness of imaging with respect to clinical practice guidelines. However, the high proportion of imaging use across 12-months, particularly with the exclusion of people with serious pathology in this cohort, calls into question the appropriateness of imaging. The appropriateness of current imaging use for older adults is unknown. Assessing the appropriateness of imaging in older populations in future studies is important to see whether interventions to reduce inappropriate imaging are also possible for older adults.

## Conclusion

Three out of five older adults with LBP who sought chiropractic care received imaging over one-year. Participants receiving imaging tended to have more complex presentations (e.g., more disability, suspected underlying pathology) or stronger beliefs that imaging was necessary for the management of LBP. Degenerative changes were the most common imaging finding. Pathology of potential clinical relevance was present on approximately 15% of imaging reports received. No conditions requiring immediate medical attention were reported.

## Electronic supplementary material

Below is the link to the electronic supplementary material.

Supplementary Material 1

## Data Availability

The datasets used and/or analysed during the current study are available from the corresponding author on reasonable request.

## Abbreviations

LBP: Low back pain
BACE:C-A: BAck Complaints in Elders: Chiropractic-Australia
CT: Computed tomography
MRI: Magnetic resonance imaging
DXA: Dual energy x-ray absorptiometry
