# Supplementary material for: Diagnostic imaging in the management of older adults with low back pain: analysis from the BAck Complaints in Elders: Chiropractic – Australia cohort study
Source: Chiropr Man Therap. 2024 Dec 18;32:40. doi: 10.1186/s12998-024-00562-z (PMC11656767; doi:10.1186/s12998-024-00562-z)
Supplement: Supplementary file 1 — Supplementary Material 1 [file 12998_2024_562_MOESM1_ESM.docx]

Additional file Table 1: Characteristics of chiropractors involved in the BACE:C-A study

| Chiropractor characteristics (n=28) |  |
| --- | --- |
| Female | 46% |
| Age (median, range) | 43, 27-65 |
| Years in clinical practice (median, range) | 16, 3-38 |
| Practice location (Australian state or territory)* |  |
| NSW | 43% |
| VIC | 21% |
| WA | 18% |
| QLD | 14% |
| TAS | 7% |
| Radiography facilities or referral |  |
| In-house | 11% |
| Medical radiologist | 89% |
| Imaging frequency for adults 55 years or older |  |
| Occasionally | 61% |
| Half of the time | 18% |
| Very often | 14% |
| Always | 7% |
| There is a role for the use of lumbar spine imaging (x-rays, CT or MRI) in the evaluation of patients aged ≥55 years with acute low back pain (less than 3 months duration), even in the absence of red flags for serious disease |  |
| Agree | 43% |
| Neutral or disagree | 57% |
| In patients aged ≥55 years, routine X-rays of the lumbar spine are recommended prior to initiating spinal manipulative therapy |  |
| Agree | 29% |
| Neutral or disagree | 71% |
| *1 chiropractor practiced in both NSW and QLD |  |

Additional file Table 2: Proportions of imaging referral by different healthcare practitioners (n, %, 95%CI)

| Timepoint | Number of people receiving imaging | Referred for imaging by general medical practitioner | Referred for imaging by chiropractor | Referred for imaging by physiotherapist | Referred for imaging by other |
| --- | --- | --- | --- | --- | --- |
| Any imaging | 132 | 62, 47.0, 38.7-55.5 | 90, 68.2, 59.8-75.5 | 6, 4.5, 2.1-9.6 | 12, 9.1, 5.3-15.2 |
| Baseline | 52 | 37, 71.2, 57.7- 81.7 | 16, 30.8, 19.9- 42.3 | 1, 1.9, 0.3- 10.1 | 6, 11.5, 5.4- 23.0 |
| 2-weeks | 13 | 2, 15.4, 4.3-42.2 | 2, 15.4, 4.3-42.2 | 2, 15.4, 4.3-42.2 | 0, 0.0, 0.0-22.8 |
| 6-weeks | 19 | 11, 57.9, 36.3-76.9 | 8, 42.1, 23.1-63.7 | 0, 0.0, 0.0-16.8 | 1, 5.3, 0.9-24.6 |
| 3-months | 10 | 5, 50.0, 23.7-76.3 | 3, 30.0, 10.8-60.3 | 2, 20.0, 5.7-51.0 | 0, 0.0, 0.0-27.8 |
| 6-months | 69 | 3, 4.3, 1.5-12.0 | 41, 59.4, 47.6-70.2 | 0, 0.0, 0.0-5.3 | 2, 2.9, 0.8-10.0 |
| 9-months | 16 | 12, 75.0, 50.5-89.8 | 2, 12.5, 3.5-36.0 | 1, 6.2, 1.1-28.3 | 2, 12.5, 3.5-36.0 |
| 12-months | 15 | 9, 60.0, 35.8-80.2 | 3, 20.0, 7.1-45.2 | 0, 0.0, 0.0-20.4 | 4, 26.7, 10.9-52.0 |

Proportions along each row do not add up to 100%. Some participants had multiple imaging referrals and were referred by different healthcare practitioners. Some participants did not report who referred them for imaging.

Additional file Table 3: Baseline characteristics of the participants in the BACE-C:A cohort (N=132) who received imaging, stratified by those that provided their imaging report (N=58) and those that did not (N=74).

| Characteristic | Participants receiving imaging who provided an imaging report | Participants receiving imaging who did not provide an imaging report |
| --- | --- | --- |
| Age (mean, SD) | 67.1, 7.8 | 67.2, 7.6 |
| Sex (n, %, 95%CI) |  |  |
| Male | 29, 50.0, 37.5-62.5 | 39, 54.2, 42.7-65.2 |
| Female | 29, 50.0, 37.5-62.5 | 33, 45.8, 34.8-57.3 |
| Unspecified | - | - |
| Low back pain intensity (mean, SD)* | 6.24, 2.4 | 5.3, 2.2 |
| Duration of low back pain (n, %, 95%CI)* |  |  |
| Less than 6 weeks | 20, 34.5, 22.8-48.2 | 41, 56.2, 44.1-67.5 |
| 6 weeks to 3 months | 10, 17.2, 9.0-29.9 | 7, 9.6, 4.3-19.3 |
| More than 3 months | 28, 48.3, 35.1-61.7 | 25, 34.2, 23.8-46.4 |
| Low back disability (mean, SD) | 8.0, 5.0 | 6.6, 5.2 |
| Previous low back pain (n, %, 95%CI) | 47, 81.0, 69.1-89.1 | 68, 93.2, 84.9-97.0 |
| Previous healthcare for low back pain (n, %, 95%CI) | 37, 63.8, 50.0-75.7 | 38, 51.4, 39.5-63.0 |
| Pain extending below the knee (n, %, 95%CI) | 15, 25.9, 15.6-39.2 | 15, 20.3, 12.2-31.5 |
| Serious cause of low back pain (n, %, 95%CI) | 28, 48.3, 35.1-61.7 | 29, 39.2, 28.3-51.3 |
| Past history of cancer (n, %, 95%CI) | 4, 8.7, 3.4-20.3 | 11, 19.0, 10.9-30.9 |
| Taking glucocorticoid medication (n, %, 95%CI)^#^ | 1, 1.7, 0.3-9.1 | 2, 2.8, 0.8- 9.6 |
| X-rays or scans are necessary to get the best medical care for low back pain (n, %, 95%CI) | | |
| Agree | 44, 75.9, 62.5-85.7 | 46, 63.9, 51.7-74.6 |
| Neutral | 7, 12.1, 5.4-24.0 | 20, 27.8, 18.2-39.8 |
| Disagree | 7, 12.1, 5.4-24.0 | 6, 8.3, 3.4-17.9 |
| Everyone with low back pain should have spine imaging (e.g. X-ray, CT, or MRI) (n, %, 95%CI) | | |
| Agree | 38, 65.5, 51.8-77.2 | 36, 50.0, 38.7-61.3 |
| Neutral | 12, 20.7, 11.6-33.7 | 25, 34.7, 24.1-46.9 |
| Disagree | 8, 13.8, 6.6-26.0 | 11, 15.3, 8.2-26.1 |

*statistically significant difference in this variable between those that provided imaging reports and those that did not, p<0.05; ^#^p-value not reported, data too sparse

Additional file Table 4: Proportion of categories of imaging findings stratified by the individual and the type of imaging report (n, %, 95%CI)

|  | Findings per individual (n=58) | Findings per imaging report (n=71) | Findings per x-ray report (n=29) | Findings per CT report (n=22) | Findings per MRI report (n=15) | Findings per bone scan report (n=2) | Findings per DXA report (n=3) |
| --- | --- | --- | --- | --- | --- | --- | --- |
| **Congenital anomaly** | 6, 10.3, 4.8-20.8 | 7, 9.9, 4.9-19.0 | 1, 3.5, 0.6-17.2 | 4, 18.2, 7.3-38.5 | 2, 13.3, 3.7-37.9 | 0, 0.0, 0.0-65.8 | 0, 0.0, 0.0-56.2 |
| Transitional segment | 4, 6.9, 2.7-16.4 | 5, 7.0, 3.1-15.5 | 1, 3.5, 0.6-17.2 | 3, 13.6, 4.8-33.3 | 1, 6.7, 1.2-29.8 | 0, 0.0, 0.0-65.8 | 0, 0.0, 0.0-56.2 |
| Block vertebra | 0, 0.0, 0.0-6.2 | 0, 0.0, 0.0-5.1 | 0, 0.0, 0.0-11.7 | 0, 0.0, 0.0-14.9 | 0, 0.0, 0.0-20.4 | 0, 0.0, 0.0-65.8 | 0, 0.0, 0.0-56.2 |
| Other | 4, 6.9, 2.7-16.4 | 4, 5.6, 2.2-13.6 | 1, 3.5, 0.6-17.2 | 2, 9.1, 2.5-27.8 | 1, 6.7, 1.2-29.8 | 0, 0.0, 0.0-65.8 | 0, 0.0, 0.0-56.2 |
| **Spondylolisthesis** | 18, 31.0, 20.6-43.8 | 20, 28.2, 19.0-39.5 | 3, 10.3, 3.6-26.4 | 7, 31.8, 16.4-52.7 | 10, 66.7, 41.7-84.8 | 0, 0.0, 0.0-65.8 | 0, 0.0, 0.0-56.2 |
| Isthmic | 6, 10.3, 4.8-20.8 | 6, 8.5, 3.9-17.2 | 1, 3.5, 0.6-17.2 | 1, 4.5, 0.8-21.8 | 4, 26.7, 10.9-52.0 | 0, 0.0, 0.0-65.8 | 0, 0.0, 0.0-56.2 |
| Degenerative | 9, 15.5, 8.4-26.9 | 10, 14.1, 7.8-24.0 | 1, 3.5, 0.6-17.2 | 6, 27.3, 13.2-48.2 | 3, 20.0, 7.1-45.2 | 0, 0.0, 0.0-65.8 | 0, 0.0, 0.0-56.2 |
| Other | 4, 6.9, 2.7-16.4 | 4, 5.6, 2.2-13.6 | 1, 3.5, 0.6-17.2 | 3, 13.6, 4.8-33.3 | 0, 0.0, 0.0-20.4 | 0, 0.0, 0.0-65.8 | 0, 0.0, 0.0-56.2 |
| **Alignment anomaly** | 27, 46.6, 34.3-59.2 | 30, 42.3, 31.5-53.9 | 14, 48.3, 31.4-65.6 | 10, 45.5, 26.9-65.3 | 6, 40.0, 19.8-64.3 | 0, 0.0, 0.0-65.8 | 0, 0.0, 0.0-56.2 |
| Other alignment change (pelvic tilt, lordosis etc) | 12, 20.7, 12.3-32.7 | 12, 16.9, 9.9-27.3 | 3, 10.3, 3.6-26.4 | 5, 22.7, 10.1-43.4 | 4, 26.7, 10.9-52.0 | 0, 0.0, 0.0-65.8 | 0, 0.0, 0.0-56.2 |
| *Scoliosis* | 18, 31.0, 20.6-43.8 | 21, 29.6, 20.2-41.0 | 12, 41.4, 25.5-59.3 | 6, 27.3, 13.2-48.2 | 3, 20.0, 7.1-45.2 | 0, 0.0, 0.0-65.8 | 0, 0.0, 0.0-56.2 |
| Degenerative | 3, 5.2, 1.8-14.1 | 3, 4.2, 1.5-11.7 | 2, 6.9, 1.9-22.0 | 0, 0.0, 0.0-14.9 | 1, 6.7, 1.2-29.8 | 0, 0.0, 0.0-65.8 | 0, 0.0, 0.0-56.2 |
| Other scoliosis | 16, 27.6, 17.8-40.2 | 18, 25.4, 16.7-36.6 | 10, 34.5, 19.9-52.7 | 6, 27.3, 13.2-48.2 | 2, 13.3, 3.7-37.9 | 0, 0.0, 0.0-65.8 | 0, 0.0, 0.0-56.2 |
| **Arthritis** | 56, 96.6, 88.3-99.1 | 65, 91.6, 82.8-96.1 | 26, 89.7, 73.6-96.4 | 22, 100.0, 85.1-100.0 | 15, 100.0, 79.6-100.0 | 2, 100.0, 34.2, 100.0 | 0, 0.0, 0.0-56.2 |
| Disc degeneration | 44, 75.9, 63.5-85.0 | 50, 70.4, 59.0-79.8 | 22, 75.9, 57.9-87.8 | 13, 59.1, 38.7-76.7 | 15, 100.0, 79.6-100.0 | 0, 0.0, 0.0-65.8 | 0, 0.0, 0.0-56.2 |
| Facet degeneration | 44, 75.9, 63.5-85.0 | 50, 70.4, 59.0-79.8 | 14, 48.3, 31.4-65.6 | 21, 95.5, 78.2-99.2 | 13, 86.7, 62.1-96.3 | 2, 100.0, 34.2, 100.0 | 0, 0.0, 0.0-56.2 |
| DISH | 1, 1.7, 0.3-9.1 | 1, 1.4, 0.3-7.6 | 1, 3.5, 0.6-17.2 | 0, 0.0, 0.0-14.9 | 0, 0.0, 0.0-20.4 | 0, 0.0, 0.0-65.8 | 0, 0.0, 0.0-56.2 |
| Inflammatory arthritis | 0, 0.0, 0.0-6.2 | 0, 0.0, 0.0-5.1 | 0, 0.0, 0.0-11.7 | 0, 0.0, 0.0-14.9 | 0, 0.0, 0.0-20.4 | 0, 0.0, 0.0-65.8 | 0, 0.0, 0.0-56.2 |
| Other degenerative | 14, 24.1, 15.0-36.5 | 16, 22.5, 14.4-33.5 | 10, 34.5, 19.9-52.7 | 4, 18.2, 7.3-38.5 | 0, 0.0, 0.0-20.4 | 2, 100.0, 34.2, 100.0 | 0, 0.0, 0.0-56.2 |
| **Disc lesion** | 34, 58.6, 45.8-70.4 | 37, 52.1, 40.7-63.3 | 0, 0.0, 0.0-11.7 | 22, 100.0, 85.1-100.0 | 15, 100.0, 79.6-100.0 | 0, 0.0, 0.0-65.8 | 0, 0.0, 0.0-56.2 |
| Bulge | 32, 55.2, 42.5-67.3 | 35, 49.3, 38.0-60.7 | 0, 0.0, 0.0-11.7 | 22, 100.0, 85.1-100.0 | 13, 86.7, 62.1-96.3 | 0, 0.0, 0.0-65.8 | 0, 0.0, 0.0-56.2 |
| Herniation | 6, 10.3, 4.8-20.8 | 6, 8.5, 3.9-17.2 | 0, 0.0, 0.0-11.7 | 2, 9.1, 2.5-27.8 | 4, 26.7, 10.9-52.0 | 0, 0.0, 0.0-65.8 | 0, 0.0, 0.0-56.2 |
| Annular tear/fissure | 3, 5.2, 1.8-14.1 | 3, 4.2, 1.5-11.7 | 0, 0.0, 0.0-11.7 | 0, 0.0, 0.0-14.9 | 3, 20.0, 7.1-45.2 | 0, 0.0, 0.0-65.8 | 0, 0.0, 0.0-56.2 |
| Other | 0, 0.0, 0.0-6.2 | 0, 0.0, 0.0-5.1 | 0, 0.0, 0.0-11.7 | 0, 0.0, 0.0-14.9 | 0, 0.0, 0.0-20.4 | 0, 0.0, 0.0-65.8 | 0, 0.0, 0.0-56.2 |
| **Ligamentum flavum hypertrophy** | 10, 17.2, 9.6-28.9 | 10, 14.1, 7.8-24.0 | 0, 0.0, 0.0-11.7 | 5, 22.7, 10.1-43.4 | 5, 33.3, 15.2-58.3 | 0, 0.0, 0.0-65.8 | 0, 0.0, 0.0-56.2 |
| **Spinal stenosis** | 29, 50.0, 37.5-62.5 | 32, 45.1, 34.1-56.6 | 1, 3.5, 0.6-17.2 | 17, 77.3, 56.6-89.9 | 14, 93.3, 70.2-98.8 | 0, 0.0, 0.0-65.8 | 0, 0.0, 0.0-56.2 |
| Central | 19, 32.6, 22.1-45.6 | 21, 29.6, 20.2-41.0 | 1, 3.5, 0.6-17.2 | 12, 54.6, 34.7-73.1 | 853.3, 30.1-75.2 | 0, 0.0, 0.0-65.8 | 0, 0.0, 0.0-56.2 |
| Lateral | 26, 44.8, 32.8-57.6 | 29, 40.9, 30.2-52.5 | 0, 0.0, 0.0-11.7 | 15, 68.2, 47.3-83.6 | 14, 93.3, 70.2-98.8 | 0, 0.0, 0.0-65.8 | 0, 0.0, 0.0-56.2 |
| **Modic change/ Bone oedema** | 8, 13.8, 7.2-24.9 | 8, 11.3, 5.8-20.7 | 0, 0.0, 0.0-11.7 | 1, 4.5, 0.8-21.8 | 7, 46.7, 24.8-69.9 | 0, 0.0, 0.0-65.8 | 0, 0.0, 0.0-56.2 |
| Modic changes type 1 | 3, 5.2, 1.8-14.1 | 3, 4.2, 1.5-11.7 | 0, 0.0, 0.0-11.7 | 0, 0.0, 0.0-14.9 | 3, 20.0, 7.1-45.2 | 0, 0.0, 0.0-65.8 | 0, 0.0, 0.0-56.2 |
| Modic changes type 2 | 3, 5.2, 1.8-14.1 | 3, 4.2, 1.5-11.7 | 0, 0.0, 0.0-11.7 | 0, 0.0, 0.0-14.9 | 3, 20.0, 7.1-45.2 | 0, 0.0, 0.0-65.8 | 0, 0.0, 0.0-56.2 |
| Modic changes type 3 | 0, 0.0, 0.0-6.2 | 0, 0.0, 0.0-5.1 | 0, 0.0, 0.0-11.7 | 0, 0.0, 0.0-14.9 | 0, 0.0, 0.0-20.4 | 0, 0.0, 0.0-65.8 | 0, 0.0, 0.0-56.2 |
| Endplate oedema | 4, 6.9, 2.7-16.4 | 4, 5.6, 2.2-13.6 | 0, 0.0, 0.0-11.7 | 1, 4.5, 0.8-21.8 | 3, 20.0, 7.1-45.2 | 0, 0.0, 0.0-65.8 | 0, 0.0, 0.0-56.2 |
| Bone marrow oedema | 1, 1.7, 0.3-9.1 | 1, 1.4, 0.3-7.6 | 0, 0.0, 0.0-11.7 | 0, 0.0, 0.0-14.9 | 1, 6.7, 1.2-29.8 | 0, 0.0, 0.0-65.8 | 0, 0.0, 0.0-56.2 |
| **Trauma** | 7, 12.1, 6.0-22.9 | 8, 11.3, 5.8-20.7 | 2, 6.9, 1.9-22.0 | 3, 13.6, 4.8-33.3 | 3, 20.0, 7.1-45.2 | 0, 0.0, 0.0-65.8 | 0, 0.0, 0.0-56.2 |
| Compression fracture, new, traumatic | 0, 0.0, 0.0-6.2 | 0, 0.0, 0.0-5.1 | 0, 0.0, 0.0-11.7 | 0, 0.0, 0.0-14.9 | 0, 0.0, 0.0-20.4 | 0, 0.0, 0.0-65.8 | 0, 0.0, 0.0-56.2 |
| Compression fracture, old traumatic/osteoporosis | 6, 10.3, 4.8-20.8 | 7, 9.9, 4.9-19.0 | 2, 6.9, 1.9-22.0 | 2, 9.1, 2.5-27.8 | 3, 20.0, 7.1-45.2 | 0, 0.0, 0.0-65.8 | 0, 0.0, 0.0-56.2 |
| Compression fracture, new pathology/osteoporosis | 1, 1.7, 0.3-9.1 | 1, 1.4, 0.3-7.6 | 0, 0.0, 0.0-11.7 | 1, 4.5, 0.8-21.8 | 0, 0.0, 0.0-20.4 | 0, 0.0, 0.0-65.8 | 0, 0.0, 0.0-56.2 |
| Other | 1, 1.7, 0.3-9.1 | 1, 1.4, 0.3-7.6 | 0, 0.0, 0.0-11.7 | 0, 0.0, 0.0-14.9 | 1, 6.7, 1.2-29.8 | 0, 0.0, 0.0-65.8 | 0, 0.0, 0.0-56.2 |
| **Osteoporosis** | 5, 8.6, 3.7-18.6 | 6, 8.5, 3.9-17.2 | 1, 3.5, 0.6-17.2 | 3, 13.6, 4.8-33.3 | 0, 0.0, 0.0-20.4 | 0, 0.0, 0.0-65.8 | 2, 66.7, 20.8-93.9 |
| Osteopenia | 4, 6.9, 2.7-16.4 | 4, 5.6, 2.2-13.6 | 0, 0.0, 0.0-11.7 | 2, 9.1, 2.5-27.8 | 0, 0.0, 0.0-20.4 | 0, 0.0, 0.0-65.8 | 2, 66.7, 20.8-93.9 |
| Osteoporosis | 0, 0.0, 0.0-6.2 | 0, 0.0, 0.0-5.1 | 0, 0.0, 0.0-11.7 | 0, 0.0, 0.0-14.9 | 0, 0.0, 0.0-20.4 | 0, 0.0, 0.0-65.8 | 0, 0.0, 0.0-56.2 |
| Possible osteoporosis | 1, 1.7, 0.3-9.1 | 2, 2.8, 0.8-9.7 | 1, 3.5, 0.6-17.2 | 1, 4.6, 0.8-21.8 | 0, 0.0, 0.0-20.4 | 0, 0.0, 0.0-65.8 | 0, 0.0, 0.0-56.2 |
| **Serious pathology*** | 5, 8.6, 3.7-18.6 | 5, 7.0, 3.1-15.5 | 1, 3.5, 0.6-17.2 | 2, 9.1, 2.5-27.8 | 2, 13.3, 3.7-37.9 | 0, 0.0, 0.0-65.8 | 0, 0.0, 0.0-56.2 |
| Tumour | 3, 5.2, 1.8-14.1 | 3, 4.2, 1.5-11.7 | 0, 0.0, 0.0-11.7 | 2, 9.1, 2.5-27.8 | 1, 6.7, 1.2-29.8 | 0, 0.0, 0.0-65.8 | 0, 0.0, 0.0-56.2 |
| Infection | 0, 0.0, 0.0-6.2 | 0, 0.0, 0.0-5.1 | 0, 0.0, 0.0-11.7 | 0, 0.0, 0.0-14.9 | 0, 0.0, 0.0-20.4 | 0, 0.0, 0.0-65.8 | 0, 0.0, 0.0-56.2 |
| Other | 3, 5.2, 1.8-14.1 | 3, 4.2, 1.5-11.7 | 1, 3.5, 0.6-17.2 | 0, 0.0, 0.0-14.9 | 2, 13.3, 3.7-37.9 | 0, 0.0, 0.0-65.8 | 0, 0.0, 0.0-56.2 |
| **Pathology or trauma of likely clinical relevance** | 9, 15.5, 8.4-26.9 | 10, 14.1, 7.8-24.0 | 2, 6.9, 1.9-22.0 | 4, 18.2, 7.3-38.5 | 2, 13.3, 3.7-37.9 | 0, 0.0, 0.0-65.8 | 2, 66.7, 20.8-93.9 |
| **Soft tissue** | 6, 10.3, 4.8-20.8 | 7, 9.9, 4.9-19.0 | 2, 6.9, 1.9-22.0 | 3, 13.6, 4.8-33.3 | 1, 6.7, 1.2-29.8 | 1, 50.0, 9.5-90.6 | 0, 0.0, 0.0-56.2 |
| **Prior surgery** | 3, 5.2, 1.8-14.1 | 3, 4.2, 1.5-11.7 | 1, 3.5, 0.6-17.2 | 2, 9.1, 2.5-27.8 | 0, 0.0, 0.0-20.4 | 0, 0.0, 0.0-65.8 | 0, 0.0, 0.0-56.2 |

*****Pathology included three haemangiomas (tumour), two Tarlov cysts (other), and one femoral neck bone infarct (other). One participant had both a haemangioma and a Tarlov cyst
